# Management of cellulitis and the role of the nurse: a 5-year retrospective multicentre study in Fako, Cameroon

**DOI:** 10.1186/s13104-019-4497-4

**Published:** 2019-07-23

**Authors:** Bachi-Ayukokang Ebob-Anya, Nahyeni Bassah, John Ngunde Palle

**Affiliations:** 1 Department of Nursing, Faculty of Health Sciences, University of Buea, P.O Box 63, Buea, South-West Region Cameroon; 2 Saint Luke’s Medical Center Buea, Buea, Cameroon

**Keywords:** Cellulitis management, Nursing care, Cameroon

## Abstract

**Objective:**

This was a 5 year retrospective study of patients’ hospital records to find out how patients with cellulitis are managed and the care provided by nurses to these patients in some hospitals in Fako, Cameroon.

**Results:**

Of the 236 cases of cellulitis identified from a study of hospital records, 202 were included in the study. Most of the participants (55%) were female and the mean (SD) age was 43 (1.1) years. Cellulitis accounted for 2.3% of admissions in this study. The predisposing factors identified were; the presence of trauma (60.5%), HIV infection (18.6%), alcohol consumption (8.4%) and tobacco use (4.8%). Commonly recorded complications were necrosis (32.2%), sepsis (23%), abscess formation (19.5%), and ulcer development (19.5%). Medical management was with antibiotic therapy, including mostly penicillin (26.5%), aminoglycoside (22.1%), nitroimidazole (20.2%) and cephalosporin (19.6%). Debridement (46.7%), and incision and drainage (44.4%) were the most implemented surgical interventions. Nursing care, as found in patients’ hospital records were predominantly on medication administration (98.0%), vital signs assessment (90.5%) and patient assessment (53%). Cellulitis therefore was found among a substantial number of patients and management was predominantly with combination antibiotics therapy and inadequate nursing care.

**Electronic supplementary material:**

The online version of this article (10.1186/s13104-019-4497-4) contains supplementary material, which is available to authorized users.

## Introduction

Cellulitis, a common medical emergency and cause of infection-related hospital admission worldwide, varies in severity from mild to life threatening [1, 2]. It is a non-necrotizing inflammation of the skin and subcutaneous tissue usually caused by Staphylococcus or Streptococcus infection that does not involve the muscle or fascia [3]. It can occur anywhere on the body but its occurrence on the lower limbs is more frequent. There are various risk factors associated with lower extremities cellulitis such as lymphedema, leg edema, venous insufficiency, traumatic wound, leg ulcers, toe-wed intertrigo, and excoriated leg dermatosis [1, 4–7]. Patients with cellulitis may present either at the Out Patient Department (OPD), general medical, elderly care, dermatology, orthopedic, vascular or general surgery departments [8].

## Main text

### Study context and study population

In Cameroon in 2017, Njim et al. [9] found the disruption of the skin barrier and the presence of toe-web intertrigo to be strongly associated with lower limb cellulitis. Moreover, in 2015, cellulitis accounted for 1447 disability adjusted life years (DALYs) in Cameroon and affected mostly men and members in the lower class of society [9, 10]. It is also associated with a prolonged hospital stay, with resultant reduction in income and economic productivity, and consequent worsening of the vicious cycle of poverty [9–11]. The burden of cellulitis has been envisaged to increase in subsequent years in Africa due to the presence of factors such as increasing prevalence of obesity and voluntary depigmentation of the skin [11]. In Cameroon, patients with cellulitis are mostly admitted to the surgical unit, with critical cases in the intensive care unit, if available. Those with less severe infections, could be consulted on an outpatient basis, but will require regular outpatient visits for follow up care, as there are no formalized home care services in Cameroon. Moreover, there are currently no guidelines on cellulitis management and treatment is based on the attending healthcare providers’ judgment. Nurses in Cameroon play a vital role in patient care in the hospitals as they are the largest group of health professionals and spend more time with patients. They, like other nurses globally, carryout nursing roles like caregiving, communication, patient advocacy, counselling and teaching. Nonetheless, there is a dearth of published literature on the management of cellulitis in Cameroon, and the role played by nurses in this management. This paper therefore presents findings from a study of patients’ hospital records that sought to find out how patients with cellulitis are managed and the care provided by nurses to these patients in some hospitals in Fako, Cameroon.

### Methods

The study was carried out in the Fako division in Cameroon, which is one of the six divisions of the South-West Region. This region has an estimated population of 1,534,232 (2015) inhabitants and covers a total area of approximately 25,410 km^2^ [12]. This study was carried out in three healthcare facilities including; one government owned regional hospital, a Cameroon Development Corporation owned Hospital, and a lay private primary health center, specialized in orthopedic care. These health facilities are geographically accessible to the local population.

The study was a retrospective hospital-based cohort study of patients admitted over a 5 year period, from March 2013 to February 2018. Participants included patients admitted to the surgical department of the three selected health services with a primary diagnosis of cellulitis. The sampling strategy was consecutive and exhaustive. The hospital’s admission registers were first reviewed and patients with a primary diagnosis of cellulitis, within the study period, were identified. The hospital files of the identified patients were then sorted out from the hospital store and reviewed. The nursing shifts reports for these patients were also identified and studied. Patients with incomplete or missing files were excluded from further analysis.

Data were collected using a researcher-designed check-list, consisting of three sections. “Introduction” section sort information on demographic data and risk factors like: age, sex, religion, ethnic group, marital status, history of alcohol consumption and tobacco use, site of cellulitis infection, phase of infection at consultation (grouped as ‘At Onset’ which is a period of less than 6 days between apparition of signs and consultation Or ‘At later phase’ which is above 1 week.) use of self-prescribed medications, systemic symptoms, recurrent infection and presence of a co-morbidity. Section two was on management approaches including medical, and surgical management as well as on nursing care relating to the caregiving, communication, patient advocacy, teaching and counselling roles of the nurse. Nurses’ caregiving roles included general patient assessment, vital signs assessment (regular-daily monitoring in the morning, afternoon and evening periods or irregular-omissions of recordings of at least 6 periods), drug administration, hygiene and elimination care, patient feeding pain management, wound care and ambulatory care. Section three was on patient outcome (recorded complications, length of hospital stay and response to treatment).

Data were analyzed using both descriptive (frequency count, percentage and mean) and inferential statistics, with the aid of SPSS version 21. The Chi square test was used to compare phase of infection at consultation with change in regimen for significant difference. Findings were presented on frequency distribution tables and on charts. All statistics were set at 95% confidence interval.

### Results

All three hospitals had a total of 10,306 admissions during the study period, and 236 of these admissions were cases of cellulitis. Thus cellulitis accounted for 2.3% of admissions in all facilities combined. However, only 202 patients’ files (85.6%) were included in further analysis, as they were complete. The files of 34 (14.4%) patients were either incomplete or missing, and thus were excluded. Of the 202 patient studied, 111 (55.0%) were female, and 91 (45.0%) males. The mean (SD) age of the participants was 43 (1.1), with range from 1 to 85 years. These participants were from diverse occupational groups but mostly consisted of laborers of the Cameroon Development Corporation (24.3%), farmers (16.3%), traders (8.4%), students (6.1%) and housewife (6.1%) (Table 1). Trauma (60.5%), HIV (18.6%), alcohol consumption (8.4%) and tobacco use (4.8%) were most predominant predisposing factors (Table 2).

**Table 1:** Demographic data of cellulitis patients

Demographic information	Test statistics	Statistical values	
	Categories	Frequency	Percentage (%)
Age in categories	0–14	6	3.0
	15–24	10	5.0
	25–44	102	50.5
	45–64	63	31.2
	65+	21	10.4
	Total	202	100.0
Sex	Female	111	55.0
	Male	91	45.0
	Total	202	100.0
Occupation	CDC laborer	49	24.3
	Local farmer	33	16.3
	Business	17	8.4
	Student	13	6.1
	House wife	13	6.1
	Jobless	12	5.9
	Retired	11	5.4
	Tapper	7	3.5
	Driver	6	3
	Not recorded	16	7.9
	Others	25	12.5
	Total	202	100

**Table 2:** Other Risk factors associated with the development of cellulitis

Predisposing factors	Responses N	Percentage (%)
Trauma	101	60.5
HIV	31	18.6
Alcohol	14	8.4
Tobacco	8	4.8
Carbuncle	4	2.4
Obesity	3	1.8
Existing ulcer	2	1.2
Athlete foot	1	0.6
Dental origin	1	0.6
Steroid induced cushing syndrome	1	0.6
Malnutrition	1	0.6
Total	167	100.1

The highest number of admissions occurred in the month of December with a total of 26 cases (12.9%), followed by August [23 (11.4%)], July [21 (10.4%)], and November [20 (9.9%)]. Admissions were least common in the month of May, with only 8 admissions. A steady increase was seen in number of cases from year 2 to 5 (Additional file 1). Seventy-six (37.6%) patients consulted at onset of their infection, while 70 (34.7%) consulted at the later phase. Fifty-six (27.7%) case files had no indication on when signs and symptoms started. Thirty-three (16.3%) of the patients had consulted previously before their contact with the study hospitals. The lower limb was the most common lesion site, with 188 (93.0%) of cases. Eight (4.0%) of the patients had cellulitis on their upper limb and 6 (3.0%) on the face. Up to 52 (25.7%) cases presented with one or more systemic symptoms such as fever, headache and lymphadenitis. A few of the patients (12.4%) received self-prescribed medications such as antibiotics, analgesics and traditional remedies before consultation. Moreover, 17 (8.4%) cases experienced a reoccurrence of infection. Sixty-seven (33.2%) cases had their infection associated with complications such as necrosis (32.2%), sepsis (23%), abscess formation (19.5%), ulcer (19.5%), deep vein thrombosis (3.4%), pussy pustules (1.1%) and lymphangitis (1.1%). Complete recovery was registered for 178 (88.1%) patients while fourteen (6.9%) were discharged against medical advice, 5 (2.5%) were referred, 3 (1.5%) died and 2 (1.0%) absconded from the hospital. The mean (SD) length of hospital stay was 11.2 (0.7) days, with a range from 1 to 74 days.

All patients were placed on antibiotic therapy, with 30 (15%) on mono-antibiotic therapy and 172 (85%) on combination therapy. Out of those placed on combination therapy, 114 patients (56.4%) were placed on 2 antibiotics, 52 (25.7%) placed on 3 antibiotics, 5 (2.5%) were placed on 4 antibiotics and 1 (0.5%) placed on 5 antibiotics. Penicillin (26.5%), aminoglycoside (22.1%), nitro imidazole (20.2%) and cephalosporin (19.6%) were the most common antibiotics used (Additional file 2). Ninety-one patients (45%) had their antibiotic regimen changed along the course of treatment, 62.6% of who were those who consulted at the onset of infection while 37.4% where those who consulted at a later phase. The mean (SD) duration of the antibiotic regimen was 9.47 (0.4) days.

Forty-five (19.8%) patients underwent surgical interventions, these included wound debridement 21 (46.7%), abscess incision and drainage 20 (44.4%), skin grafting 3 (6.7%) and 1 amputation (2.2%) (Additional file 3). One hundred and seven (53.0%) patients had their health history assessed and recorded by a nurse. The vital signs for 99 (49.0%) patients were regularly checked and that for 94 (46.5%) patients were irregularly assessed and recorded. Twenty-three patients (11.4%) had cold compress applications and 198 (98.0%) had their medications administered and recorded in their files. Generally, there was almost no record of patient education in the files. However, in 2.5% of files patients were reported to have received education about their nutrition (Table 3).

**Table 3:** Care provided and recorded by nurses

Nursing role	Nursing interventions	Recorded	Number of cases	Percentage (%)
Care giver	History taking and physical assessment	Yes	107	53
		No	95	47
		Total	202	100
	Vital signs assessment	Regularly	99	49
		Irregularly	94	46.5
		Never	9	4.5
		Total	202	100
	Care provision		Number of cases	Percentage (%)
	Application of a cold compress		23	11.4
	Nursing care plan		2	1
	Medication administration		198	98
Educator	Patient education and area		Number of cases	Percentage (%)
	Nutritional education		5	2.5
	Prevention of recurrent infection		1	0.5

### Discussion

Cellulitis was found among a substantial number of patients in the study setting. Nonetheless, the incidence is lower when compared to the study of Concheiro et al. in Western Australia who recorded 8.6% [13]. This difference could be attributed to the fact that their study was carried out in a more specialized dermatology department which narrows down the number of admissions whereas our study was done in the surgical unit. In line with other studies [8, 9, 13] our study revealed a higher female to male ratio, which was not the case with previous studies [1, 14, 15]. In our setting, women are more likely to be involved in farming where they are exposed to injuries that predisposes them to cellulitis. Cellulitis was also common among young adults and this is consistent with a previous report [15]. Young adults make a majority of the work force population hence increasing the possibility of trauma and consequent infection. Moreover, most of those affected in this study were local farmers and laborers of the Cameroon Development Corporation, who are predominantly young adults, and who do not have adequate protective tools like gloves and booths. Trauma, was therefore a common risk factor of cellulitis in this study as in previous studies [8, 9, 11, 13, 16].

The patients in this study were most likely to visit these healthcare facilities after having consulted elsewhere or taken self prescribed medications, like NSAIDs, antibiotics or traditional remedies [8, 11, 13]. It is worth noting that these hospitals serve as reference hospitals for surgical cases in this setting. Interestingly, the percentage of patients who had complications was small compared to the perception of development of life threatening complications as stated in literature [14].

A majority of the patients in our study were placed on combination therapy, similar to the previous reports by Aly et al. [17]. Almost half of the patients had their regimen changed, and this was fourfolds higher than the value reported by Dong et al. [18]. Surprisingly, a majority of the patients with regimen change were those who came to the hospital at the onset of the infection. The reason for this is not quite evident. Nevertheless, we noted that the mean duration of antibiotics use in this study is longer than the recommended 5 days by the Infectious Disease Society of America 2014 guidelines for the diagnosis and management of skin and soft tissue infection [19].

Generally, nurses were seen to play a limited role in the management of cellulitis in these health facilities, as was found in patients’ hospital records and nurses’ shift reports. The role of the nurse in health assessment includes obtaining a history and performing a physical assessment [20]. Nurses need to be able to recognize the manifestation of cellulitis so as to rule out other conditions and commence therapy immediately [21]. In line with this, a good number of cases were assessed by nurses upon admission and throughout their hospital stay. On the other hand, only few patients received fundamental nursing care, and this was limited to medication administration and application of a cold compress. This practice is contrary to the use of a warm compress as recent literature states [20]. As concerns education, the number of patients who were educated is negligible. The failure of nurses to get a firm grip on this aspect of care renders patients unaccustomed to their practices [22]. Educating patients can assist recovery and adherence to treatment. Generally, most expected nursing roles were either left undone or unrecorded.

Documentation is a daily reality of nurses’ work as it gives evidence to the care rendered by nurses and hence negligence of this role is one of the causes of the existence of alternative knowledge in nursing practice [23]. The implementation and documentation of the nursing process in all patient-nurse care encounters is an ideal that has challenged the nursing profession and discipline [24]. In conjunction with this, whether the nurses in these health facilities did render high standards of care or not, we could not fully appreciate their work due to inadequate documentation.

In conclusion there was an integrated management of cellulitis, however, this was marked by use of antibiotics combination therapy, and inadequate nursing care provision.

## Limitations

Our study was carried out in the surgical units and did not include the out-patient and intensive care units hence the exact incidence for these hospitals is not reflected in our study. Moreover, this study was conducted in only three hospitals in Fako, limiting the possibility of generalizing the findings to the entire population of Fako. In addition, due to poor documentation, our study could not fully identify predisposing factors, and the role of the nurse. Thus these results should be taken with caution.

## Additional files

**Additional file 1.** Figure showing distribution of cellulitis cases per year per month.

**Additional file 2.** Figure showing antibiotics administered to patients.

**Additional file 3.** Table showing the types of surgical managent recieved by patients.

## Data Availability

The datasets used during the current study are available from the corresponding author on reasonable request.

## Abbreviations

HIV: human immunodeficiency virus
OPD: out patient department
DALY: disability adjusted life years
SPSS: statistical package for social sciences
SD: standard deviation
NSAID: non-steroidal anti-inflammatory drug
