# Supplementary material for: Management of cellulitis and the role of the nurse: a 5-year retrospective multicentre study in Fako, Cameroon
Source: BMC Res Notes. 2019 Jul 23;12:452. doi: 10.1186/s13104-019-4497-4 (PMC6651919; doi:10.1186/s13104-019-4497-4)
Supplement: Supplementary file 1 — Additional file 1. Figure showing distribution of cellulitis cases per year per month. [file 13104_2019_4497_MOESM1_ESM.docx]

Figure 1: Distribution of number of Cellulitis cases per year per month

Figure 1: Distribution of number of Cellulitis cases per year per month
