# Supplementary material for: Management of cellulitis and the role of the nurse: a 5-year retrospective multicentre study in Fako, Cameroon
Source: BMC Res Notes. 2019 Jul 23;12:452. doi: 10.1186/s13104-019-4497-4 (PMC6651919; doi:10.1186/s13104-019-4497-4)
Supplement: Supplementary file 2 — Additional file 2. Figure showing antibiotics administered to patients. [file 13104_2019_4497_MOESM2_ESM.docx]

**Figure 2: Antibiotics administered to patients**
