# Supplementary material for: Management of cellulitis and the role of the nurse: a 5-year retrospective multicentre study in Fako, Cameroon
Source: BMC Res Notes. 2019 Jul 23;12:452. doi: 10.1186/s13104-019-4497-4 (PMC6651919; doi:10.1186/s13104-019-4497-4)
Supplement: Supplementary file 3 — Additional file 3. Table showing the types of surgical managent recieved by patients. [file 13104_2019_4497_MOESM3_ESM.docx]

**Table 4: Types of surgical interventions undergone by study participants**

| **Surgical intervention** | **Number of patients** | **Percentage (%)** |
| --- | --- | --- |
| Debridement | 21 | 46.7 |
| Incision and drainage | 20 | 44.4 |
| Skin grafting | 3 | 6.7 |
| Amputation | 1 | 2.2 |
| **Total** | **45** | **100.0** |
